# Effective treatment of acute graft-versus-host disease following liver transplantation using an integrated regimen centered on antithymocyte globulin: a single-center experience

**DOI:** 10.3389/fimmu.2026.1696936

**Published:** 2026-02-03

**Authors:** Qiming Zheng, Li Zhang, Honghai Wang, Ababakri Abdisamat, Xiaodong Wang, Xia Xiao, Wentao Jiang

**Affiliations:** 1 Department of General Surgery/Transplantation, First Central Hospital of Tianjin Medical University, Tianjin, China; 2 Department of Liver Transplantation, Tianjin First Central Hospital, Tianjin, China; 3 Tianjin Key Laboratory of Molecular Diagnosis and Treatment of Liver Cancer, Tianjin First Center Hospital, Tianjin, China; 4 Tianjin Key Laboratory for Organ Transplantation, Tianjin First Center Hospital, Tianjin, China; 5 Department of Hematology, Tianjin First Central Hospital, Tianjin, China; 6 Institute of Transplantation Medicine, Nankai University, Tianjin, China

**Keywords:** acute graft-versus-host disease, antithymocyte globulin, diagnosis, immunosuppression, liver transplantation, treatment

## Abstract

**Aims:**

Acute graft-versus-host disease (aGVHD) after liver transplantation (LT) is a rare but highly lethal complication, with no standardized diagnosis/treatment protocols. Our center successfully treated three patients using a comprehensive antithymocyte globulin (ATG)-based regimen. This study preliminarily evaluates the efficacy of ATG-based regimens for aGVHD, comparing outcomes with a historical cohort that received conventional treatment, to provide initial clinical evidence.

**Methods:**

This retrospective study included three consecutive post allogeneic LT aGVHD patients treated at Tianjin First Central Hospital between January 2024 and August 2025. Their outcomes were compared with those of 10 historical controls who received conventional treatment between January 2019 and December 2023. Given the small sample size, outcomes were primarily compared descriptively and using effect size measures (odds ratio [OR]/median difference), while Fisher’s exact and Mann–Whitney *U* tests were performed for exploratory purposes.

**Results:**

In the ATG treatment group, two patients presented with fever and rash, while one presented with diarrhea; all developed skin involvement, oral ulcers, and myelosuppression, with a median posttransplant onset of 18 days (range: 15–27). Skin biopsy revealed epidermal hyperkeratosis, basal cell vacuolar degeneration, and dermal lymphocytic infiltration. Cases 2 and 3 had donor CD8+ T-cell chimerism. Median onset-to-diagnosis time was 15 days (range: 9–17). Treatment included an ATG-based regimen, tacrolimus tapering/withdrawal, low-dose glucocorticoids, intravenous immunoglobulin (IVIG), janus kinase (JAK) inhibitor (ruxolitinib), and symptomatic supportive care. All three patients achieved complete remission (CR) without serious complications, with a median diagnosis-to-remission time of 35 days (range: 17–50). In this preliminary comparison, the ATG group demonstrated a significantly higher CR rate (100% *vs*. 20%; *p* = 0.012, OR = 25.0), a lower infection rate (0% *vs*. 60%; *p* = 0.033), and a shorter median remission time (35 days *vs*. 62 days; *p* = 0.021, median difference = − 27 days) compared with historical controls.

**Conclusion:**

The diagnosis of aGVHD after LT requires integrating clinical manifestations, rash pathology, and chimerism detection. In this small series, ATG-based therapy was associated with favorable outcomes compared with a historical cohort receiving conventional therapy, suggesting that it may be a promising strategy warranting further evaluation in large-scale clinical studies.

## 1 Introduction

Graft-versus-host disease (GVHD) is defined as an immune attack by functionally competent donor-derived immune cells against the immunosuppressed recipient (1). Following liver transplantation (LT), GVHD is a rare but highly fatal complication. Its pathogenesis involves donor-derived reactive T lymphocytes recognizing recipient alloantigens, which trigger excessive immune responses and subsequent immunopathological damage to multiple organs (e.g., skin, gastrointestinal tract, bone marrow) (1). A recent systematic review of 130 publications, covering 225 patients with post-LT GVHD from 1988 to 2020, reported an incidence of 1.2% and an overall mortality rate of 71% (2). GVHD is categorized by onset timing into acute (occurring within 100 days posttransplant) and chronic forms, with acute GVHD (aGVHD) being the clinically predominant subtype. Notably, early diagnosis of aGVHD remains challenging, and its prognosis is extremely poor.

Clinically, aGVHD following LT primarily involves the skin, oral cavity, gastrointestinal tract, and bone marrow. Typical manifestations include fever, rash, oral ulcers, diarrhea, and pancytopenia—symptoms that are often misdiagnosed or delayed due to their nonspecificity (2). Definitive diagnosis primarily relies on histopathological examination of biopsies from affected organs (3), with donor lymphocyte infiltration and characteristic morphological changes in tissues providing supplementary evidence (4). Chimerism, defined as the presence of donor-derived cells (particularly T lymphocytes) in the recipient, also serves as key auxiliary evidence for diagnosis (5). These donor-derived immune cells can recognize recipient tissues as foreign and initiate an immune attack, thereby triggering aGVHD—an association further supported by the detection of donor cells in the recipient’s peripheral blood or tissues. Notably, unlike aGVHD associated with hematopoietic stem cell transplantation (HSCT), the donor liver is an “immunologically privileged organ”. Consequently, this type of aGVHD is primarily mediated by donor-derived T cells rather than hematopoietic stem cell (HSC) chimerism. Therefore, CD8+ T-cell chimerism detection offers higher specificity for diagnosing aGVHD following LT. Despite these insights, widely accepted clinical or laboratory diagnostic criteria remain lacking, and no consensus has been reached regarding the optimal treatment regimen for aGVHD following LT.

Traditional intensified immunosuppression offers no survival benefit and may increase the risk of infection (2), whereas reducing or withdrawing immunosuppression can restore the patient’s immune system and improve outcomes (6). Nevertheless, scattered case reports and small retrospective studies consistently document treatment failures, indicating that current regimens are suboptimally effective. Notably, almost all existing treatments for LT-associated aGVHD are extrapolated from single-center experiences with aGVHD following HSCT. These reported protocols typically involve high-dose glucocorticoids ± intravenous immunoglobulin (IVIG) ± reduction or discontinuation of calcineurin inhibitors (CNIs), but their efficacy remains unsatisfactory (2). Given the uncertainty and high mortality of LT-associated aGVHD, the targeted therapeutic approach investigated in this study holds significant clinical value. Antithymocyte globulin (ATG) exerts immunosuppressive effects by targeting T-cell surface antigens and depleting pathogenic T lymphocytes (including CD4+ subsets) (7); thus, this study adopted an ATG-centered comprehensive intervention strategy. The regimen includes ATG, tapering or discontinuation of the immunosuppressant tacrolimus, low-dose glucocorticoids, IVIG, janus kinase (JAK) inhibitors (ruxolitinib), and symptomatic supportive care.

Based on this, we retrospectively analyzed the clinical features, diagnostic process, and therapeutic course of three LT patients with aGVHD who were successfully treated with this protocol. To further evaluate the efficacy of the ATG-based combination regimen, we conducted a historical comparison with patients who had previously received conventional aGVHD therapy. By comparing outcomes between the two groups, we aimed to explore diagnostic and therapeutic strategies for post-LT aGVHD and provide clinical evidence to improve patient prognosis.

## 2 Materials and methods

### 2.1 Study design and patient enrollment

This study is a retrospective, controlled analysis. Clinical data were collected from three patients who developed aGVHD following allogeneic LT at Tianjin First Central Hospital between January 2024 and August 2025. The analysis examined their clinical characteristics and treatment experiences in detail. A retrospective comparative analysis was conducted using a historical control group comprising 10 patients with aGVHD following LT who received conventional treatment regimens (glucocorticoid pulse therapy ± IVIG ± CNI dose adjustment) at this center between January 2019 and December 2023. All patients in the historical control group met diagnostic criteria for aGVHD. Patients with other severe organ failure, malignant tumor recurrence, or loss to follow-up were excluded. This study aims to evaluate the efficacy and safety of a comprehensive treatment regimen centered on ATG by comparing the prognosis of two patient groups. All patients in the ATG group and surviving patients in the historical control group provided written informed consent. For deceased control group patients, the ethics committee approved an exemption from informed consent. This study was approved by the Medical Ethics Committee of Tianjin First Central Hospital.

### 2.2 Perioperative immunosuppressive therapy regimen for LT

During LT, patients received intravenous methylprednisolone sodium succinate (10 mg/kg) and an interleukin (IL)-2 receptor antagonist (IL-2RA; basiliximab, 20 mg) prior to portal vein anastomosis (or hepatic reperfusion).

After LT, patients received triple immunosuppressive therapy consisting of oral tacrolimus, mycophenolic acid analogs, and low-dose methylprednisolone (administered intravenously initially, followed by oral transition). Tacrolimus trough blood concentrations were maintained at 5–10 ng/mL. Oral mycophenolate mofetil (MMF) 360–540 mg twice daily was initiated on postoperative day 4. Methylprednisolone sodium succinate was administered intravenously at 100, 80, 60, 40, and 20 mg once daily on postoperative days 1 to 5, respectively; it was then switched to oral methylprednisolone 10 mg/day on postoperative day 6, reduced to 8 mg/day on day 7, further reduced to 4 mg/day at 1 month postoperatively, and discontinued at 3 months postoperatively.

### 2.3 Multidisciplinary teamwork

In view of the rarity and lethality of aGVHD after LT, our center has implemented multidisciplinary consultation for all suspected cases, involving specialties such as hematology, dermatology, infectious diseases, LT, and rheumatology. The core principles for diagnosis and treatment are as follows: Clinical features—including disease onset timing, and the morphology and distribution of skin lesions—are highly suggestive of aGVHD; skin biopsy should be performed immediately if a rash is present, and bone marrow biopsy should be conducted promptly if pancytopenia occurs. ATG is the cornerstone of treatment and should be combined with low-dose hormones, IVIG, immunosuppressant tapering or withdrawal, and JAK inhibitors. Meanwhile, intensive anti-infective therapy must be administered. In addition, myelosuppression should be alleviated as soon as possible, and blood purification therapy may be applied to mitigate the hyperactive immune response.

### 2.4 Diagnostic methods for aGVHD

**Figure 1:**
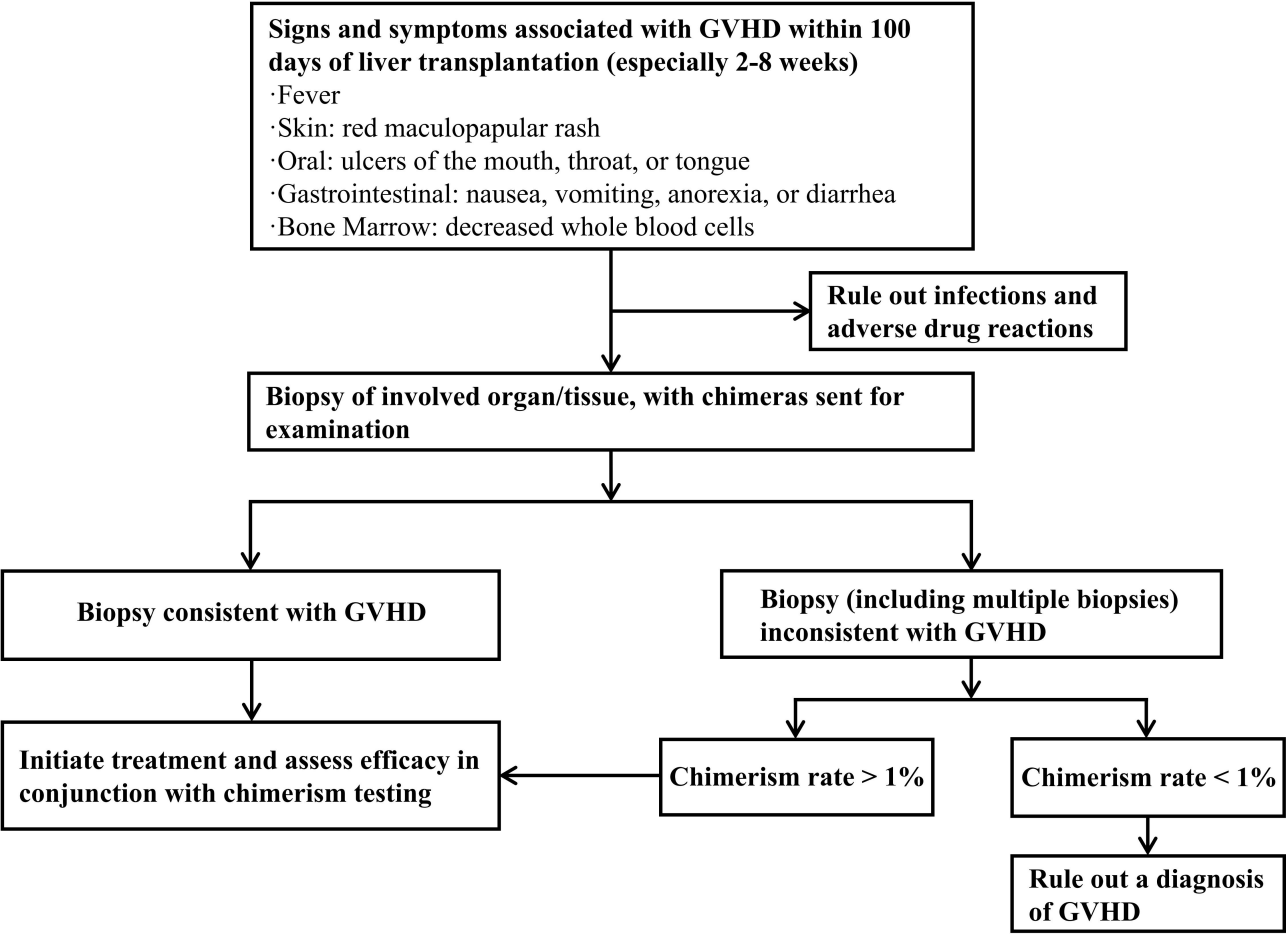
Diagnostic process for aGVHD after LT.

There are currently no fully standardized diagnostic criteria for post-LT GVHD. This study adopted a widely used multimodal diagnostic model, which includes the following: clinical manifestations of target organ involvement (such as fever, rash, oral ulcers, gastrointestinal symptoms, and bone marrow suppression); typical histopathological features of skin or bone marrow biopsy; and chimerism rate test results of donor-derived CD8 T cells (detection method described in **Supplementary File 1**). Given the critical role of early diagnosis and intervention in improving prognosis, patients with unexplained fever, rash, diarrhea, or pancytopenia within 100 days after LT (especially 2–8 weeks postoperatively) should be highly alert to the possibility of aGVHD. Relevant biopsies should be performed promptly after ruling out infections and adverse drug reactions. Once the diagnosis is confirmed by biopsy, treatment should be initiated immediately. The diagnostic process for aGVHD in this study is detailed in **Figure 1**.

### 2.5 Treatment for aGVHD

For patients with highly suspected or confirmed GVHD, strict isolation and protection measures are immediately implemented, including single-room isolation, intensive indoor air disinfection, the use of laminar flow beds, and the assignment of dedicated nurses to provide respiratory and gastrointestinal protective care. Pharmacological treatment adheres to the principles of both etiological and symptomatic therapy:

1. Etiological treatment: Immunosuppressants (tacrolimus, with monitoring of blood concentrations) are reduced or discontinued, and low-dose glucocorticoid maintenance therapy (1–2 mg/kg/day), ATG (1 mg/kg/day for 3–5 days), JAK inhibitors (ruxolitinib: 10 mg bid), and IVIG (10 g/day) are administered concurrently.
2. Symptomatic and supportive therapy: Includes broad-spectrum anti-infection prophylaxis (caspofungin for antifungal prophylaxis, acyclovir for antiviral prophylaxis, cefoperazone/sulbactam for antibacterial prophylaxis), correction of granulocyte deficiencies (recombinant human granulocyte-stimulating factor [rhG-CSF], with individualized dosage), and nutritional and metabolic support (antidiarrheals, diuretics, glucose monitoring, etc.).

For the historical control group, the conventional treatment regimen was as follows: methylprednisolone 5–10 mg/kg/day for 3–5 days as pulse therapy, combined with IVIG 0.4 g/kg/day for 5 consecutive days, with dosage adjustment of CNIs as necessary.

### 2.6 Criteria for aGVHD complete remission

Assessment of aGVHD remission following LT requires a multidimensional, dynamic evaluation that incorporates resolution of clinical symptoms, normalization of laboratory parameters, ability to discontinue immunosuppressive agents, and long-term relapse-free survival. No single universal criterion has been established to date. Given that aGVHD predominantly involves the skin, mucous membranes, gastrointestinal tract, bone marrow, and liver, the core criteria for complete remission (CR) include the following elements of sustained stability (1): complete resolution of target organ symptoms, such as fever, rash, diarrhea, oral ulcers, and bone marrow suppression (2); restoration of complete blood count (CBC) to the normal reference range (3); reduction of CD8+ T-lymphocyte chimerism levels from peripheral blood donors to < 1%, maintained for at least 2 weeks, during which no recurrence of aGVHD symptoms should occur. It is important to note that, based on the principle of nonmaleficence in medical ethics and the evidence from Akbulut et al. (1), who demonstrated that 89% of patients with aGVHD-related rash exhibited a significant correlation between complete clinical resolution and histological improvement (characterized by the resolution of basal cell vacuolization and the reduction/disappearance of dermal lymphocytic infiltration), repeated histological biopsy is not mandatory in posttreatment patients who do not explicitly consent to the procedure.

### 2.7 Statistical analysis

Continuous variables were presented as mean ± standard deviation (SD) or median (range), depending on the results of the Shapiro–Wilk normality test. Variables following a normal distribution (e.g., recipient age) were analyzed using the independent-samples *t*-test, while nonnormal variables (e.g., remission time, *p* < 0.05) were analyzed using Mann–Whitney *U* test. Categorical variables were presented as counts (percentages) and compared using Fisher’s exact test (due to small sample size). To quantify clinical relevance, effect size measures were calculated: odds ratios (OR) and 95% confidence intervals (CIs) for categorical variables, and median differences (95% CIs) for continuous variables. Consistent with clinical consensus, an OR > 10 was defined as a large effect size, and continuous variables with 95% CIs that did not cross 0 were considered clinically meaningful.

Given the extremely small sample size (*n* = 3) and retrospective nature of the ATG group, the statistical power of this exploratory study—conducted in the context of rare diseases—is extremely low. This results in wide 95% CIs and limited reliability of the statistical estimates. Furthermore, no formal statistical comparison of baseline characteristics between the two groups was performed. Therefore, the *p*-values presented are for exploratory purposes only and should not be interpreted as confirmatory evidence of efficacy. Results should be interpreted with emphasis on effect sizes and clinical relevance rather than statistical significance. All *p*-values and effect sizes are highly susceptible to individual patient variability and should be treated with caution. No adjustments for multiple comparisons were applied to preserve statistical power in this exploratory context, although this increases type I error. Analyses were conducted using SPSS 26.0 (IBM Corporation, Armonk, NY, USA), with two-sided *p*-values < 0.05 considered statistically significant.

## 3 Results

All three patients with post-LT aGVHD were men, with a median age of 56 years (range: 54–69). Donor–recipient ABO blood groups were compatible, and two cases had a donor–recipient age difference of more than 20 years. Primary diseases included two cases of hepatocellular carcinoma (HCC) complicated by hepatitis B cirrhosis, and one case of primary biliary cirrhosis (PBC) complicated by esophageal variceal bleeding (EGVB). Previous treatments included transcatheter arterial chemoembolization (TACE) in two patients, one of whom also underwent radiofrequency ablation (RFA) for a small HCC located at the liver dome; the remaining patient had no prior surgical history. Regarding comorbidities, one patient had a history of both type 2 diabetes mellitus and hypertension. Detailed baseline characteristics of the patients are summarized in **Table 1**.

**Table 1:** Baseline characteristics of three patients with aGVHD after LT and their corresponding donors.

Basic characteristics	Case 1	Case 2	Case 3
Recipient			
Sex	Male	Male	Male
Age (years)	54	56	69
Blood type (ABO/Rh)	A+/Rh+	A+/Rh+	B+/Rh+
Etiology	HBV, HCC, liver cirrhosis	HBV, HCC, liver cirrhosis	EGVB, PBC
Previous liver-directed therapy	Yes	Yes	No
Pre-LT treatment	TACE	TACE, RFA	–
MELD score	16	7	18
Child–Pugh score	B	B	C
Hypertension	−	+	−
Diabetes	−	+	−
Date of LT	29 February 2024	21 January 2025	08 March 2025
Type and mode of transplantation	Allogeneic OLT	Allogeneic OLT	Allogeneic OLT
Donor			
Sex	Female	Male	Male
Age (years)	24	69	44
Blood type (ABO/Rh)	A+/Rh+	A+/Rh+	B+/Rh+
Age difference between donor and recipient (years)	30	13	25

*LT*, liver transplantation; *HBV*, hepatitis B virus; *HCC*, hepatocellular carcinoma; *EGVB*, esophageal variceal bleeding; *PBC*, primary biliary cirrhosis; *TACE*, transarterial chemoembolization; *RFA*, radiofrequency ablation; *OLT*, orthotopic liver transplantation; “−”, no/none; “+”, yes/present; “↓”, reduction.

**Figure 2:**
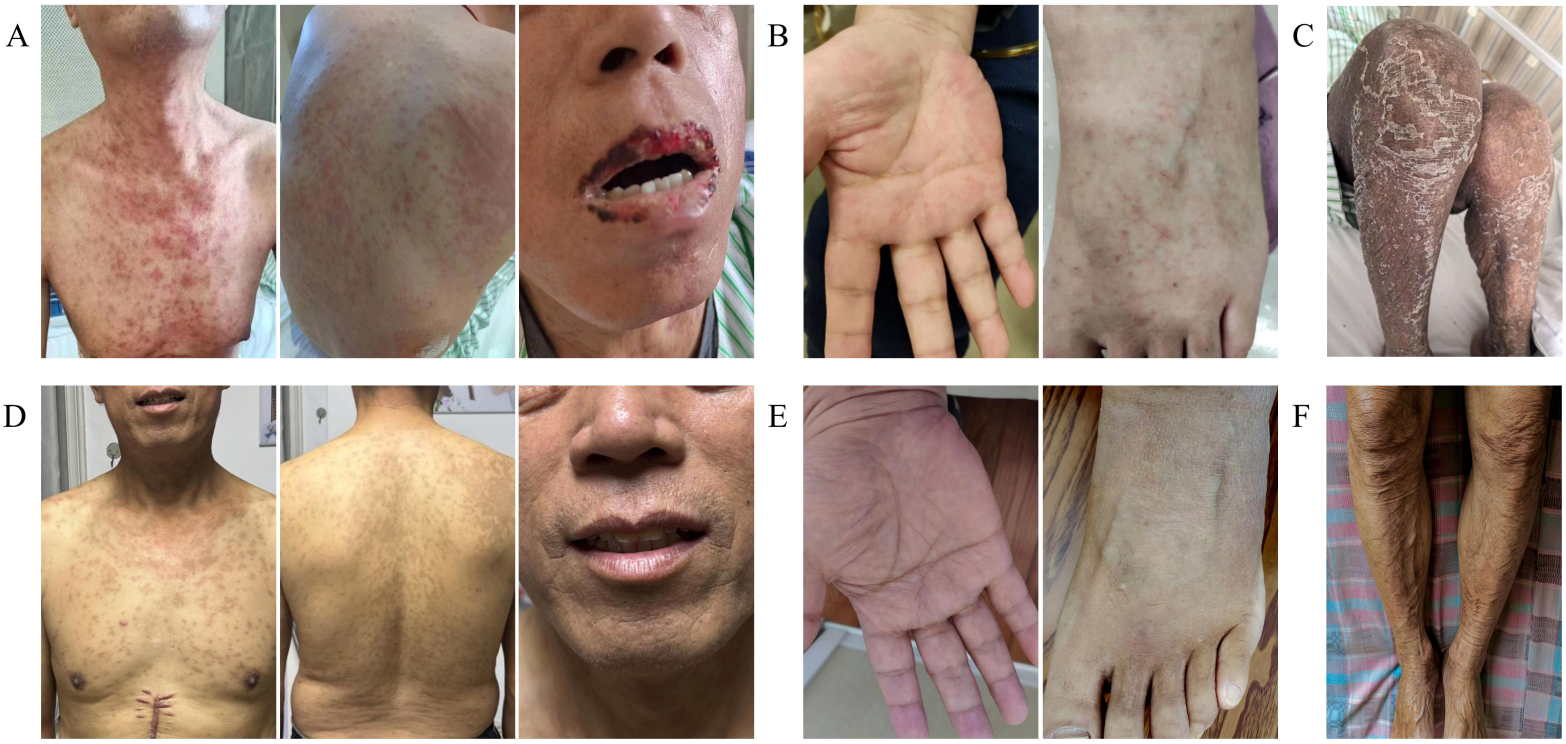
Skin rash manifestations in three patients with aGVHD following LT before and after treatment. **(A)** Case 1: Red macules on the anterior chest and posterior back, with localized fusion of the macules into patches, accompanied by generalized flushing of the skin, accompanied by exfoliation of the epidermis of the oral mucous membranes, and ulceration of the skin. **(B)** Case 2: Punctate red maculopapular rash distributed on the palms of the hands and the backs of the feet. **(C)** Case 3: Extensive rash and peeling of both lower extremities. **(D–F)** Significant improvement and resolution of rashes were observed in three patients after treatment with ATG-based therapy.

**Figure 3:**
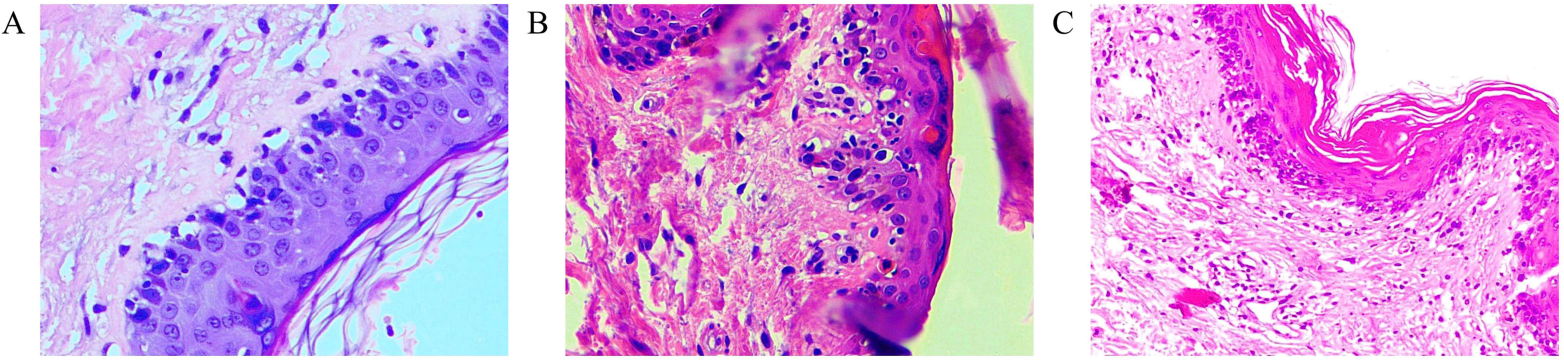
Skin biopsy results of three patients with aGVHD after LT (H&E, × 100). **(A)** Case 1: Thinning of the epidermis, focal vacuolization of basal cells, occasional poorly keratinized epithelial cells within the epidermis, scattered vacuolization of epidermal cells, scattered infiltration of a few lymphoid cells and eosinophils within the epidermis, and infiltration of predominantly inflammatory cells with lymphocytes around the small blood vessels of the superficial dermis, with scattered neutrophils. **(B)** Case 2: Focal basal cell liquefaction of the epidermis with scattered dyskeratotic cells and scattered lymphocytic infiltration. **(C)** Case 3: Basal cell liquefaction is seen with multiple apoptotic vesicle formation and scattered lymphocyte-dominated inflammatory cell infiltration around small vessels in the superficial dermis.

All three patients exhibited typical clinical manifestations of aGVHD, including fever, skin rash (**Figures 2A–C**), diarrhea, oral ulcers, and pancytopenia, with a median time to onset of 18 days (range: 15–27) post-LT. The diagnosis of aGVHD was confirmed by skin rash biopsy in all three patients (**Figure 3**), and the median time from symptom onset to pathological diagnosis was 15 days (range: 9–17). The main clinical features of the patients are summarized in **Table 2**.

**Table 2:** Main clinical characteristics of three patients with aGVHD after liver transplantation.

Clinical characteristics	Case 1	Case 2	Case 3
Initial symptoms	Fever	Fever	Diarrhea
Involved systems	Bone marrow, oral mucosa, skin	Bone marrow, gastrointestinal, skin	Bone marrow, gastrointestinal, oral mucosa, skin
Fever	+	+	+
Time to first postoperative start (days)	15	27	29
Highest temperature (°C)	39.3	38.5	38.4
Skin rash	+	+	+
Time to first postoperative start (days)	29	30	26
Main body parts	Chest, back	Forearms, hands, and feet	Limbs and trunk
Myelosuppression	+	+	+
Time to first postoperative start (days)	22	31	35
Minimum WBC count (× 10^9^/L)	2.91	0.23	0.67
Minimum NEU count (× 10^9^/L)	2.13	0.01	0.3
Minimum PLT count (× 10^9^/L)	108	65	68
Minimum RBC count (× 10^12^/L)	2.54	2.3	2.13
Diarrhea	−	+	+
Time to first postoperative start (days)	−	39	18
Oral ulcer	+	−	+
Time to first postoperative start (days)	38	−	34
From LT to aGVHD (days)	15	27	18
Diagnostic tools	Inflammatory factor testing, skin biopsy	Bone marrow smear, skin biopsy, T(CD8) cell chimerism	Bone marrow smear, skin biopsy, T(CD8)-cell chimerism
From aGVHD to diagnosis (days)	17	9	15
From LT to biopsy (days)	32	35	33

*LT*, liver transplantation; *aGVHD*, acute graft-versus-host disease; “−”, mo/none; “+”, yes/present.

Following ATG-based comprehensive treatment, all three patients exhibited significant improvement in clinical symptoms, with marked resolution of skin rash and oral ulcers (**Figures 2D–F**). Moreover, the donor-derived CD8+ T-cell chimerism rate began to decrease after ATG administration and stabilized within the normal reference range 3–5 days posttreatment (see **Supplementary File 1** for the posttreatment CD8+ T-cell chimeric rate change curve). For patients with myelosuppression (cases 2 and 3), posttreatment bone marrow aspiration revealed recovered hematopoietic function without trilineage suppression, confirming histological remission of bone marrow involvement. For case 3, who had gastrointestinal involvement (watery diarrhea), posttreatment stool analysis showed normal flora (bacillus/coccus ratio: 90%/10%) and no leukocytes or erythrocytes, while abdominal imaging revealed resolution of incomplete intestinal obstruction, indirectly supporting histological improvement of the gastrointestinal mucosa. No significant complications occurred in the patients during treatment, and all patients were discharged once their clinical conditions stabilized. The median time from aGVHD diagnosis to meeting remission criteria was 35 days (range: 17–50). All three patients remained recurrence-free during follow-up (median: 7 months; range: 6–18), with no late-onset infections or organ dysfunction. Notably, no severe infections (e.g., cytomegalovirus [CMVs], fungal infections), transplant liver rejection, or secondary tumors were observed during the follow-up period, indicating that the long-term safety of the ATG combined with a low-dose glucocorticoid regimen is controllable. Patient-specific treatment regimens and prognostic outcomes are summarized in **Table 3**. Detailed diagnostic, therapeutic, and prognostic information for the three post-LT aGVHD patients in this study is provided in **Supplementary File 1**.

**Table 3:** Treatment and outcomes of three patients with aGVHD after liver transplantation.

Treatment and prognosis	Case 1	Case 2	Case 3
Treatment			
Discontinuation of immunosuppressants	+	−	−
Reduction of immunosuppressants	−	+	+
Tacrolimus	−	↓	↓
Lymphocyte-clearing drugs	ATG	ATG	ATG
Methylprednisolone	Small dose (1–2 mg/kg/day)	Small dose (1–2 mg/kg/day)	Small dose (1–2 mg/kg/day)
IVIG	+	+	+
Basiliximab	−	+	−
rhG-CSF	+	+	+
Anti-infective prophylaxis/therapy	+	+	+
Jak inhibitors (ruxolitinib)	+	+	+
Prognosis			
From aGVHD to remission (days)	17	35	50
Current status	Survival	Survival	Survival
Follow-up time after aGVHD (months)	18	7	6

*IVIG*, intravenous immunoglobulin; *rhG-CSF*, recombinant human granulocyte-stimulating factor; *aGVHD*, acute graft-versus-host disease; *ATG*, antithymocyte globulin; “−”, no/none; “+”, yes/present; “↓”, reduction.

From January 2019 to December 2023, our center enrolled 10 patients with post-LT aGVHD who were treated with conventional regimens. The cohort consisted of seven men and three women, with a mean age of 59.5 years ± 6.8 years. The median time to aGVHD onset was 31 days (range: 9–48). Treatment-related outcomes, assessed based on clinical symptoms and hematologic recovery, are summarized as follows: CR rate of 20% (2/10), infection rate of 60% (6/10), and median duration of CR of 62 days (range: 45–89). Three patients died of severe infections. Compared with the historical control group, the three patients in this study who received ATG-based combination therapy achieved a significantly higher CR rate (100% [3/3] *vs*. 20% [2/10], Fisher’s exact test, *p* = 0.012), a significantly lower infection rate (0/3 *vs*. 6/10, Fisher’s exact test, *p* = 0.033), and a significantly shorter median time to CR (35 days *vs*. 62 days, Mann–Whitney *U* test, *p* = 0.021; normality test: both groups *p* < 0.05, supporting nonparametric test). The effect size indicators further confirmed the clinical advantage of the ATG-based regimen: the OR for CR rate was 25.0 (95% CI: 1.3–480.0), and the median difference in remission time was − 27 days (95% CI: − 45 to − 9 days). Although the sample size was small, the large effect size (OR > 10) suggests that the advantage of the ATG-based regimen is clinically meaningful. No severe infections or other complications occurred in the ATG treatment group, which demonstrated a significantly improved prognosis. However, due to the small sample size (only three cases in the ATG group), the statistical power of Fisher’s exact test and the Mann–Whitney *U* test is limited, and the results should be interpreted with caution.

## 4 Discussion

GVHD results from a destructive cellular immune response to recipient tissues, triggered by the activation and clonal expansion of immunocompetent donor T lymphocytes (8). It is categorized into two subtypes: antibody-mediated and cell-mediated (9). Although donor-recipient HLA typing data were unavailable in this study, the clinical course, normal liver function, and favorable treatment response support a diagnosis of cell-mediated GVHD. The development of post-LT GVHD involves a three-stage cascade reaction: “immunosuppression-lymphocyte activation-tissue injury” (1): Recipient immunosuppression: Immune dysfunction induced by pre-transplant liver disease, surgical stress, and immunosuppressant use (2); Donor lymphocyte activation: Donor lymphocytes are activated through interaction with recipient antigen-presenting cells, initiating IL-2-dependent proliferation and T helper 1 (Th1) cell differentiation (3); Tissue damage and inflammation: Cytotoxic T cells target host tissue antigens, causing cell death and tissue dysfunction (10). Further donor lymphocyte activation is mediated by cytokines released from target host cells, forming a “cytokine-damage” cycle that triggers an inflammatory storm and ultimately GVHD (11). Additionally, destruction of the recipient’s skin, bone marrow, and mucosal epithelium exacerbates their immunocompromised state.

Previous studies have identified recipient age > 50 years, recipient–donor age gap > 20 years, recipient immunocompromise, multiorgan transplantation, autoimmune hepatitis, and alcoholic liver disease as high-risk factors for post-LT GVHD (6, 12). Therefore, close post-LT monitoring of these high-risk patients is essential for early detection, diagnosis, and treatment of GVHD. The diagnosis of aGVHD is often delayed due to nonspecific early symptoms. The three-dimensional diagnostic criteria proposed by Triulzi et al.—”target organ clinical manifestations, histopathological features, and donor cell chimerism status”—provide crucial clinical guidance (13).

In the present study, all three patients showed partial or complete aGVHD clinical manifestations. Unexplained hyperthermia (often > 38.0 °C) is a common initial symptom (2) and is highly prone to misdiagnosis as postoperative infection due to poor specificity. Rash is the most frequent symptom (incidence: 92.0%), typically presenting as an erythematous maculopapular rash that blanches on pressure, initially involving the chest and neck. The rash may spread diffusely, darken, coalesce, and be accompanied by pruritus; severe cases can resemble exfoliative dermatitis and must be differentiated from drug eruptions. Skin biopsy can aid diagnosis, although nonspecific findings require ruling out drug allergies and viral infections (14). Diarrhea usually occurs secondary to rash and fever, presenting as watery stools of variable severity. Small intestinal pathological biopsy may reveal crypt cell apoptosis, glandular abscesses, and partial intestinal mucosal loss (15). aGVHD-induced myelosuppression typically develops in the late stage and is often irreversible, characterized by a rapid decline in CBSs (leukocyte counts as low as 0.1–0.3 × 10^9^/L). It must be differentiated from drug toxicity, bacterial/fungal infections, and other etiologies. Patients often develop progressive pancytopenia leading to bone marrow failure, which impairs immune function and may result in death from secondary infections or other complications. Oral mucosa involvement is common, often concurrent with skin and gastrointestinal manifestations, and oral ulcers may serve as an early or synchronous manifestation of aGVHD. Since the liver is an “immunologically privileged organ”, the donor liver relatively lacks expression of receptor antigens, and liver function is typically unaffected. However, liver injury may occur secondarily to severe complications (e.g., infections or multiple organ failure) in advanced stages.

Histopathologic testing can determine whether donor-derived lymphocytes have invaded or damaged recipient tissues. Skin biopsy is safer and more straightforward than other biopsy methods. Typical pathological findings include vacuolar degeneration of the basal epithelial layer, epidermal lymphocytic infiltration, and necrotic eosinophilic keratinocytes. Additional features may include epidermal spongiosis, basal cell edema, keratinocyte necrosis, apoptosis, lymphocytic infiltration with satellite cell necrosis, and subepidermal fissures (16). In this study, rash biopsy findings in all three patients were highly suggestive of GVHD.

Taylor et al. explored recipient peripheral blood donor lymphocyte chimerism as a diagnostic tool for post-LT GVHD (10). Postoperative microchimerism (< 1%) within 1 week is common in liver transplant recipients and may promote immune tolerance and graft acceptance. In contrast, sustained large chimerism (1%–80%) for 3–4 weeks—defined as a higher proportion of donor lymphocytes detected in recipient tissues (e.g., peripheral blood, skin, gastrointestinal tract, bone marrow, oral mucosa)—is associated with increased GVHD risk (1). CD8+ T cells are the primary effector cells mediating host tissue damage in GVHD; therefore, high donor CD8+ chimerism is more diagnostically specific than total chimerism. Short tandem repeat (STR) PCR, targeting highly polymorphic genomic sequences, revealed high donor-derived CD8+ T-cell chimerism in all patients at presentation.

Current treatment regimens for aGVHD following LT are largely based on experiences from allogeneic HSCT. However, traditional approaches demonstrate limited efficacy in liver transplant recipients (2), underscoring the urgent need to optimize therapeutic strategies. The primary objective of aGVHD treatment is to restore immune homeostasis and block the cascade of tissue damage. Conventional regimens typically involve high-dose glucocorticoids (5–10 mg/kg/day) combined with IVIG or increased doses of CNIs (17). Nevertheless, these approaches have significant limitations: CR rates remain only 30%–40% (17), infection complications occur in up to 50% of patients (18), and mortality exceeds 80% in steroid-refractory cases (19).

The comprehensive treatment regimen centered on ATG employed in this study—low-dose glucocorticoids (1–2 mg/kg/day) + ATG + ruxolitinib—achieved significantly enhanced efficacy by precisely targeting the pathophysiological mechanisms of aGVHD. All three patients attained CR (100%), with no severe infections or metabolic disorders. The median time to remission was 35 days (range: 17–50), which is significantly shorter than previously reported outcomes and the efficacy of conventional therapies for GVHD at our institution. The regimen’s advantage stems from precise intervention in aGVHD pathophysiology, with ATG playing a pivotal regulatory role. Its therapeutic mechanism can be summarized as a triple synergistic effect:

First, activated T cells are selectively eliminated to interrupt the chain of cytotoxic damage. As a polyclonal antibody, ATG specifically recognizes and binds to T-cell surface antigens (e.g., CD3, CD4) (7, 20) and efficiently eliminates activated T lymphocytes from both donor and recipient sources. These cells serve as core effectors that mediate host tissue damage, and removing them directly halts cytotoxic attacks on target organs such as the skin, gastrointestinal tract, and bone marrow. ATG also modulates signaling pathways, including PI3K/AKT and AMPK (21), and suppresses the release of proinflammatory cytokines (e.g., IL-6, IFN-γ). By interrupting the “cytokine–tissue injury” positive feedback loop at its source, it mitigates damage associated with the inflammatory storm (22, 23). Furthermore, ATG modulates the immune checkpoint axis between PD-1 on activated CD8^+^ T cells and PD-L1 on monocytes, suppressing T-cell proliferation and granzyme-B release to enhance immunoregulatory effects (24). Second, it reactivates HSC function and improves immune deficiency. The key mechanism of aGVHD-related bone marrow suppression is the inhibitory effect of activated T cells on HSCs. ATG neutralizes this inhibitory signal, restoring the clonal proliferation and differentiation of recipient HSCs and thereby reestablishing bone marrow hematopoietic function (25). This effect not only alleviates the pancytopenia caused by aGVHD but also enhances the recipient’s ability to clear abnormal donor T cells by restoring immune function, creating a virtuous cycle of “immune reconstitution-disease remission” (26). Third, it induces donor-specific immune tolerance, reducing the risk of recurrence. ATG regulates negative selection processes within the thymus, eliminating self-reactive T-cell clones with high affinity for donor antigens, thereby establishing durable donor-specific immune tolerance (27). This immune tolerance prevents sustained donor T-cell attacks on recipient tissues while reducing infection risks associated with excessive immunosuppression, laying the foundation for improved long-term prognosis.

Other drugs in the regimen exhibited synergistic effects with ATG. As first-line agents for LT-associated GVHD (3), glucocorticoids control systemic immune-inflammatory responses via inducing lymphocyte apoptosis and exerting potent anti-inflammatory activity, thereby reducing target organ damage and improving recipient symptoms. No standardized starting dose exists, with reported doses varying. In this study, referencing HSCT-associated GVHD glucocorticoid regimens and our LT-associated GVHD clinical experience, all three patients with suspected GVHD were initially given low-dose glucocorticoids (2 mg/kg/day), followed by pathological biopsy and chimerism testing for confirmation. While awaiting diagnostic confirmation, none of the patients showed clinical improvement on maintenance low-dose glucocorticoids. Given the risk of glucocorticoid resistance, low-dose maintenance was continued postdiagnosis. When combined with glucocorticoids, IVIG directly enhances the recipient’s passive immunity, lowers the infection risk associated with high-dose glucocorticoids, and stabilizes immune status by inhibiting lymphocyte function, decreasing reticuloendothelial system phagocytosis, and suppressing inflammatory factor release (11). Additionally, its hematopoietic protective effect is particularly beneficial for GVHD-associated myelosuppression (28). However, the optimal dosage and duration of IVIG require further investigation. Ruxolitinib, a JAK inhibitor, specifically blocks the IL-6/IFN-γ-mediated JAK-STAT signaling pathway to suppress allogeneic T-cell attacks on recipient target organs (23). Unlike traditional immunosuppressants, ruxolitinib does not compromise overall antitumor immunity or increase the risk of posttransplant lymphoproliferative disorders, providing superior safety (29).

Dose adjustment of immunosuppressive agents remains a key point of contention in aGVHD treatment. This study supports a strategy of “tapering off rather than increasing doses”. While conventional wisdom suggests that increasing immunosuppressive doses can suppress donor T-cell activation, the recipient’s immune function is already severely compromised during aGVHD. Excessive immunosuppression further exacerbates the risk of infection. Our findings indicate that gradually tapering and discontinuing tacrolimus (maintaining serum concentrations at 2–3 ng/mL) can restore the recipient’s endogenous immune function, enhance clearance efficiency against abnormal donor T cells, reduce long-term immunosuppressive toxicity, and promote the establishment of immune tolerance. It is crucial to emphasize that during the tapering and discontinuation process, the risk of acute rejection must be closely monitored, and individualized monitoring and intervention plans should be established.

The use of immunosuppressive agents during treatment remains controversial. While the traditional view holds that dose escalation suppresses donor T-cell activation, recent studies suggest that reducing or discontinuing these agents may enhance the clearance of donor T cells by restoring recipient immune function. This study advocates this approach, with a rationale that extreme suppression of recipient immune function during GVHD can be alleviated by reducing or discontinuing immunosuppressants, thereby restoring and enhancing endogenous immune function and improving donor-derived T cells. Compared with dose escalation, tapering reduces long-term immunosuppressive toxicity, promotes immune tolerance, and mitigates global immunosuppression (30, 31). Notably, immunosuppressant withdrawal carries a risk of acute rejection, necessitating the implementation of a detailed monitoring and intervention protocol to ensure patient safety. In this study, tacrolimus blood levels were strictly monitored and maintained at 2–3 ng/mL during dose reduction or discontinuation.

Infection prevention serves as a crucial adjunctive measure in aGVHD management—uncontrolled sepsis remains the primary cause of mortality in these patients, while myelosuppression significantly increases the risk of opportunistic infections (32). This study implemented early prophylactic treatment with broad-spectrum antibiotics and antifungal agents for all patients, with particular emphasis on preventing CMV infection. Protective isolation measures were also adopted, contributing significantly to the absence of severe infections in all three patients.

The present study has several key limitations: first, the small sample size (three individuals) limits the reliable exclusion of individual variability from the conclusions. Second, the historical control group and study cohort differ in treatment eras, potentially introducing inconsistencies in adjunctive interventions (e.g., infection prevention, supportive care), which require cautious interpretation of group differences. Third, inherent selection bias in single-center retrospective studies restricts the generalizability of results. Fourth, as a clinical observational study, it lacks in-depth analysis of the independent mechanisms and synergistic regulatory networks of agents such as ATG and JAK inhibitors—an essential focus for future basic research. Finally, the delayed diagnosis of aGVHD remains challenging due to insufficient clinical awareness. To address these limitations, future aGVHD research should prioritize three core directions: developing early diagnostic technologies (e.g., early warning systems based on dynamic monitoring of CD8+ T-cell chimerism rates and proinflammatory cytokine profiling); deepening pathophysiological mechanisms to clarify the targets and synergistic effects of drugs such as ATG and JAK inhibitors for more precise, targeted therapies; and formulating individualized immunosuppression regimens for high-risk populations (e.g., age > 50 years, donor–recipient age difference > 20 years, concurrent autoimmune liver disease) to avoid excessive immunosuppression. Given the rarity of aGVHD, multicenter collaboration, standardized case sharing, and translational research will be critical for optimizing patient outcomes.

## 5 Conclusion

aGVHD after LT is a rare but highly lethal complication, diagnosed mainly based on typical clinical manifestations, histopathological features, and donor CD8+ T-lymphocyte chimerism. In this study, we used an ATG-based comprehensive treatment regimen that successfully treated three patients with post-LT aGVHD. Compared with our center’s historical cohort treated with conventional regimens, this ATG-centered strategy showed significant advantages in CR rate, infection safety, and remission time, initially confirming its efficacy and safety and indicating potential as a novel therapeutic option. However, larger clinical trials are required to optimize and validate the regimen. Future research should explore the pathogenesis of aGVHD and drug action mechanisms, and develop more targeted diagnostic and therapeutic strategies to improve long-term patient prognosis.

## Data Availability

The original contributions presented in the study are included in the article/supplementary material. Further inquiries can be directed to the corresponding authors.
