# Supplementary material for: Effective treatment of acute graft-versus-host disease following liver transplantation using an integrated regimen centered on antithymocyte globulin: a single-center experience
Source: Front Immunol. 2026 Feb 3;17:1696936. doi: 10.3389/fimmu.2026.1696936 (PMC12909242; doi:10.3389/fimmu.2026.1696936)
Supplement: Supplementary file 1 [file DataSheet1.docx]

**Supplementary file 1.**

1. **T(CD8) cell chimeric ratio detection method**

(1) Peripheral Blood Mononuclear Cell (PBMC) Isolation: Isolate PBMC from post-operative peripheral blood (pre-operative donor samples are not involved).

(2) CD8+ T Cell Magnetic Bead Positive Selection:

1) Centrifuge 1ml of PBMC suspension at 2000rpm for 5 minutes, discard the supernatant, resuspend the cells in 120μl PBS, and adjust the cell concentration to not exceed 10×10⁶ cells/ml. Transfer 100μl of the cell suspension to a 5ml round-bottom centrifuge tube labeled with sample ID and sorting type.

2) Add CD8 cell sorting solution at a volume of 100μl per ml of the diluted sample, gently pipette or vortex to mix, and incubate at room temperature for 3 minutes.

3) Vortex the CD8 sorting magnetic beads for 30 seconds, add them at a volume of 50μl per ml of the diluted sample, gently pipette to mix, and incubate at room temperature for 3 minutes.

4) Add 2.5ml EasySep Buffer to reach the specified volume (not exceeding the adsorption range of the magnetic stand), mix well by pipetting, place the uncovered centrifuge tube in the magnet, and incubate at room temperature for 3 minutes.

5) Lift the magnet, discard the supernatant into a new labeled 5ml round-bottom centrifuge tube, repeat the washing step twice with 2.5ml EasySep Buffer each time (incubate at room temperature for 3 minutes after each mixing, then discard the supernatant).

6) Add EasySep Buffer to adjust the sample volume to 0.3ml, pipette to resuspend cells adhering to the tube wall to the bottom, centrifuge briefly, transfer the cells to a 1.5ml centrifuge tube, and count the cells.

1. DNA Extraction: Extract genomic DNA from the sorted CD8+ T cells.
2. STR Detection: Perform short tandem repeat (STR) detection on the extracted DNA.
3. Chimeric Ratio Calculation: Calculate the chimeric ratio of donor cells in the post-operative sample based on the STR detection results.

**Quality Control:** STR testing utilizes the ABI 3730 sequencer, employing donor DNA as the positive control and recipient preoperative DNA as the negative control. Chimera rate calculations are performed using GeneMapper software, with a detection limit of 0.1% to ensure result accuracy.

1. **The primary course of treatment and prognosis of the three patients who developed acute graft-versus-host disease after liver transplantation.**

**Case 1:**

The patient returned to the hospital for follow-up on day 13 after liver transplantation (LT). On the 15th postoperative day, he developed unexplained fever (38.2°C), which was only briefly relieved by empirical anti-infective treatment. On the 22nd postoperative day, the fever persisted, accompanied by mild pancytopenia; Staphylococcus epidermidis was detected in the blood culture, and targeted antibacterial drug therapy was given, but plasma microbial macro-genomic sequencing (mNGS) during the same period did not suggest the causative microorganisms, and therefore, it was considered to be a possible non-infectious fever. On the 29th postoperative day, the fever did not subside and a rash appeared on the anterior chest and back (Fig. 2A), so a skin biopsy was performed to clarify the nature of the disease. The rash spread progressively while waiting for the results. On postoperative day 32, skin pathology results returned a high consideration of GVHD (Fig. 3A), which was isolated and protected. Inflammatory factor profile testing showed significantly elevated IL-6 levels and active inflammatory response, which was treated with anti-inflammatory and anti-infective therapy. After MDT consultation, the clinical manifestations, chimerism rate, rash biopsy results and inflammatory indexes were combined to confirm the diagnosis of aGVHD. aGVHD was immediately initiated: the baseline immunosuppressive regimen was discontinued; glucocorticoids were initially given as an initial shock, which did not result in any remission of the disease; the disease was upgraded to a combination of therapies, such as ATG, JAK inhibitor (rucolitinib) and intravenous IVIG; the complete blood count was dynamically monitored; and the infection prevention was strengthened with antiviral, anti-bacterial, and anti-viral drugs, which were used to prevent the disease. The patient's blood count was dynamically monitored; intensive anti-infective prophylaxis (antifungal, antibacterial, antiviral) was administered; recombinant human granulocyte stimulating factor was applied to correct the granulocyte deficiency; intestinal micro-ecological regulators were given to prevent bacterial disorders; and the evolution of the disease was closely observed. On the 38th postoperative day, oral mucosal ulcers appeared, and lidocaine gargle and local glucocorticoids were given for symptomatic treatment; at this time, the fever subsided, the rash stopped progressing, and there was no aggravation of myelosuppression or diarrhea; therefore, the use of ATG was discontinued, and the dose of the hormone was reduced step by step, and the monitoring of the blood picture was continued. On the 47th postoperative day, liver function abnormality appeared, and anaphylactic reaction suddenly occurred after cautiously adding a small dose of anti-rejection drugs, considering drug allergy or immune stress, and tacrolimus was discontinued after antihistamine treatment without recurrence; liver function was normalized in the follow-up examination. By the 48th postoperative day, the patient's general condition was good, the transplanted liver function was stable. The rash had subsided, accompanied by hyperpigmentation (Fig. 2D), and no signs of active acute graft-versus-host disease (aGVHD) were present. Following a comprehensive assessment confirming treatment efficacy, the patient was cleared for discharge.

**Case 2:**

The patient presented with unexplained fever on the 27th day after LT and was admitted to the hospital 3 days later with a rash on the forearms and hands and feet (Fig. 2 B). Admission examination showed mild leukopenia, normal liver and renal function, empirical anti-infective treatment and T-tube open drainage were given, while the rash was sent for pathologic biopsy. Tacrolimus blood concentration was monitored to be too high (11.8 ng/mL) on the following day, and the dosage was reduced. On postoperative day 34, severe neutrophil deficiency with leukopenia was observed, and G-CSF therapy was initiated with continuous hematological monitoring; a bone marrow biopsy at the same time suggested trilineage hematopoietic suppression (granulocyte/erythroid/megakaryocyte lineage), and G-CSF supportive therapy was continued. On postoperative day 35, the fever subsided, but there was a progressive decline in complete blood counts with elevated inflammatory factors; the chimerism rate of donor-derived CD8+ T cells was 46.5%, suggesting the need for close monitoring. Thereafter, the lymphocyte ratio increased progressively but the leukocytes did not rebound, and the diagnosis of aGVHD was confirmed by skin pathology (Fig. 3 B). On postoperative day 37, he was transferred to the hematology department with a positive indication for blood transfusion (HGB 70 g/L, Plt 109×10⁹/L), and was given washed red blood cell transfusion with 100-level laminar flow isolation protection. After exclusion of contraindications, an intensive immunomodulatory regimen was initiated: ATG 25 mg, low-dose glucocorticoids, tacrolimus tapering, rucolitinib, and IVIG, supplemented with broad-spectrum anti-infectives. On postoperative day 38, the rash was reduced, but the leukocytes decreased to 0.08×10⁹/L, and G-CSF was given again; repeat bone marrow biopsy still showed trilineage suppression, and then ATG treatment was upgraded: after dexamethasone 10mg pretreatment, ATG 75mg was combined with methylprednisolone 80mg intravenous pumping, with a total planned dose of 400mg; acid suppression, calcium supplementation, and tacrolimus were given simultaneously. Acid suppression, calcium supplementation, tacrolimus 0.5mg qd (concentration monitoring) and G-CSF support were given simultaneously. On postoperative day 39, diarrhea appeared (fecal smear: bacillus 95%/coccus 5%), and the chimerism rate decreased to 4.95 suggesting that the current treatment was effective; ATG 100mg/d×3 days was continued. ATG course was completed on postoperative day 42, and methylprednisolone 40mg q12h was adjusted. peripheral blood chimerism rate decreased to <1% on postoperative day 44. Hematopoietic function recovered on postoperative day 45, and the immunosuppressants were gradually reduced: methylprednisolone was reduced to 30 mg q12h, rucotinib was reduced to 5 mg bid, and tacrolimus was increased to 1 mg q12h (the concentration of 2.7 ng/mL was adjusted to 2 mg q12h); bone marrow picture improved significantly. Chimerism persisted at <1% on postoperative day 64, and rucotinib was discontinued and hormones were further reduced. By postoperative day 71, the patient's general condition remained favourable, with stable liver function and resolution of the rash (Fig. 2E). There was no evidence of active aGVHD, and the patient was discharged after evaluation of the effectiveness of treatment.

**Case 3:**

The patient presented with watery diarrhea on day 18 after LT and was admitted to the hospital 2 days later. Abdominal imaging was suggestive of incomplete intestinal obstruction, which resolved with antispasmodic analgesia, bowel rest, intravenous rehydration and enemas. On the 26th postoperative day, scattered erythematous rash appeared all over the body (especially on the inner side of both upper arms), without desquamation and epidermal breakage, which partially subsided after topical chloramphenicol tretinoin; however, the lower abdominal discomfort persisted, and the imaging still showed intestinal obstruction, and enemas were renewed. On the 29th postoperative day, a sudden unexplained fever and recurrence of rash on trunk and upper and lower limbs (Fig. 2C) were observed, and GVHD was suspected.Intermittent diarrhea was treated with montelukast combined with enema on the 31st postoperative day. Skin biopsy was performed on postoperative day 32, and peripheral blood testing on the following day showed a 4.95% chimerism rate of CD8⁺ T cells of donor origin (95.05% in the recipient, requiring dynamic monitoring). On postoperative day 34, the quadruple sign of fever, rash, alternating diarrhea and constipation, and oral ulcers appeared, and a verbal report of dermatopathology was consistent with GVHD changes (Fig. 3 C), and preemptive therapy was initiated: sterile laminar flow isolation protection; appropriate incremental glucocorticoids, and decreasing doses of tacrolimus (and monitoring of blood levels); and combination targeted therapy (rucotinib orally, ATG, and intravenous IVIG). On the 35th postoperative day, fever subsided and diarrhea stopped, but progressed to severe hypoplasia of whole blood cells (HGB 67g/L), which was supported by transfusion of suspended erythrocytes and G-CSF; a bone marrow biopsy confirmed that hematopoiesis was suppressed in all three lineages. Transfusion support was continued on postoperative days 36-37, and the rash subsided and leukocytes rebounded from day 38, suggesting response to initial therapy. There were no signs of GVHD activity on postoperative days 38-48, and prophylactic anti-infective and hormone tapering was gradually discontinued. Chimerism increased to 11.77% on postoperative day 48 and was transferred to hematology. On postoperative day 50, low-grade fever with oral ulcers resolved after anti-infection. On postoperative day 52, the chimerism rate increased to 59.4%, and ATG 50mg × 2 doses (days 54 and 56) was immediately administered, with simultaneous adjustment of immunosuppression (methylprednisolone 60mg qd, and continuation of rucotinib/tacrolimus). The skin lesions improved on postoperative day 58, the chimerism rate decreased to <1% on day 60, and the methylprednisolone was tapered in steps (50mg→40mg→30mg qd). On postoperative day 66 there was a sudden onset of pancytopenia (normal lymphocyte ratio), immune reconstitution syndrome was considered and IVIG 25g/d×5d shock, G-CSF, danazol, recombinant human thrombopoietin and methylprednisolone incremental dosage (70mg q12h) were given. On the 89th postoperative day, GVHD continued to resolve and hematopoietic stability, the regimen was adjusted: rucotinib was reduced to 5mg bid, glucocorticoid was converted to methylprednisolone 28mg qd and tapered, and tacrolimus was incremented to 0.5mg q12h. The patient was generally in good condition and had normal liver function. The cutaneous rash has resolved, accompanied by hyperpigmentation (Fig. 3F). There is no evidence of aGVHD. The patient declined further assessment of CD8 chimerism due to financial constraints. The comprehensive results suggest that the treatment is effective, and the patient is discharged from the hospital.

1. **The results of CD8 chimerism rate in cases 2 and 3 during diagnosis and treatment.**

**Case 2:**

**
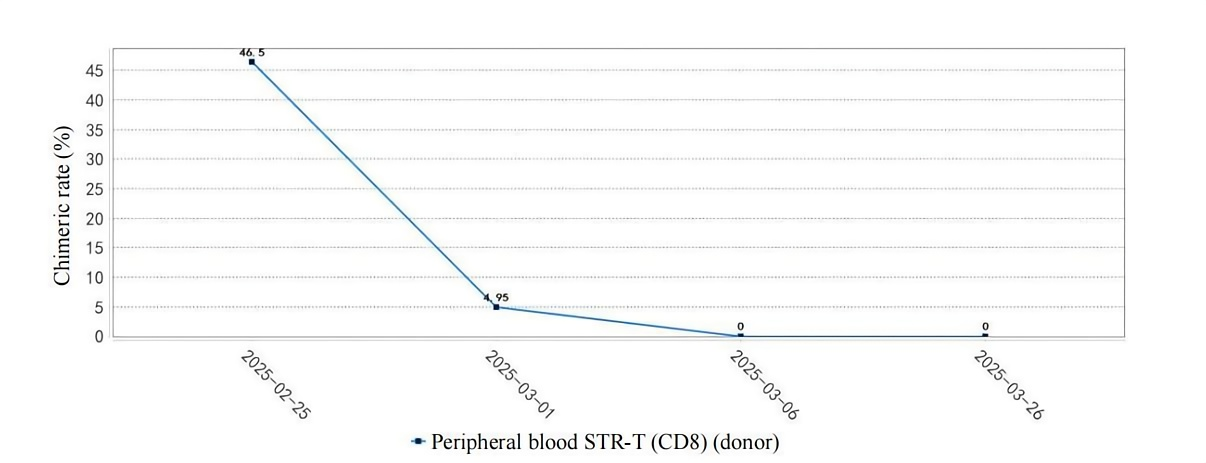
**

**Case 3:**

**
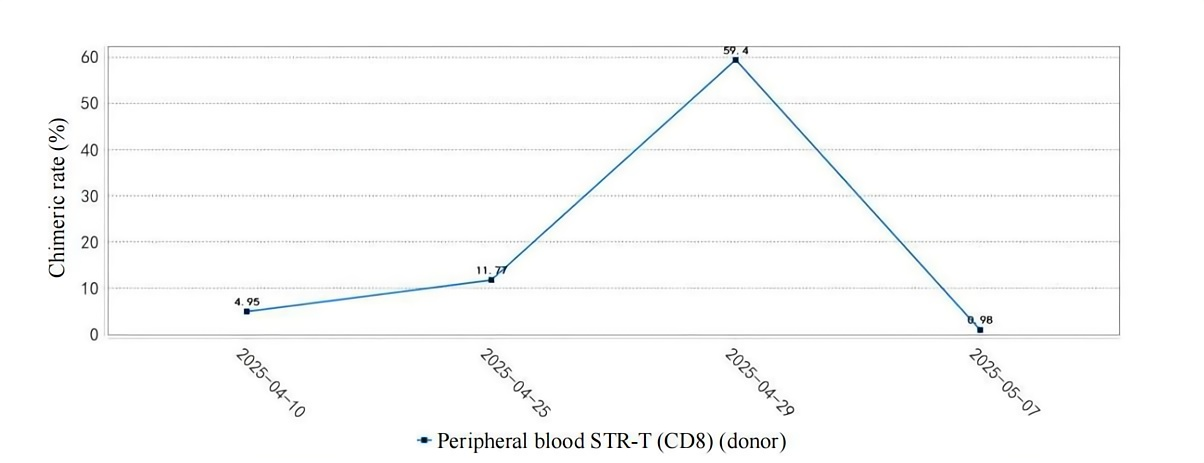
**
